# Cytotoxic and Genotoxic Effects of Cyanobacterial and Algal Extracts—Microcystin and Retinoic Acid Content

**DOI:** 10.3390/toxins13020107

**Published:** 2021-02-02

**Authors:** Michal Bittner, Alja Štern, Marie Smutná, Klára Hilscherová, Bojana Žegura

**Affiliations:** 1 RECETOX, Faculty of Science, Masaryk University, Kamenice 5, 62500 Brno, Czech Republic; michal.bittner@recetox.muni.cz (M.B.); marie.smutna@recetox.muni.cz (M.S.); klara.hilscherova@recetox.muni.cz (K.H.); 2 Department of Genetic Toxicology and Cancer Biology, National Institute of Biology, Večna pot 111, 1000 Ljubljana, Slovenia; alja.stern@nib.si

**Keywords:** cyanobacteria, algae, extracts, complex mixtures, genotoxicity, cytotoxicity, retinoic acids, microcystins, cyanotoxins, chemical analysis

## Abstract

In the last decade, it has become evident that complex mixtures of cyanobacterial bioactive substances, simultaneously present in blooms, often exert adverse effects that are different from those of pure cyanotoxins, and awareness has been raised on the importance of studying complex mixtures and chemical interactions. We aimed to investigate cytotoxic and genotoxic effects of complex extracts from laboratory cultures of cyanobacterial species from different orders (*Cylindrospermopsis raciborskii, Aphanizomenon gracile, Microcystis aeruginosa, M. viridis*, *M*. *ichtyoblabe, Planktothrix agardhii, Limnothrix redekei*) and algae (*Desmodesmus quadricauda*), and examine possible relationships between the observed effects and toxin and retinoic acid (RA) content in the extracts. The cytotoxic and genotoxic effects of the extracts were studied in the human hepatocellular carcinoma HepG2 cell line, using the MTT assay, and the comet and cytokinesis-block micronucleus (cytome) assays, respectively. Liquid chromatography electrospray ionization mass spectrometry (LC/ESI-MS) was used to detect toxins (microcystins (MC-LR, MC-RR, MC-YR) and cylindrospermopsin) and RAs (ATRA and 9cis-RA) in the extracts. Six out of eight extracts were cytotoxic (0.04–2 mg_DM_/mL), and five induced DNA strand breaks at non-cytotoxic concentrations (0.2–2 mg_DM_/mL). The extracts with genotoxic activity also had the highest content of RAs and there was a linear association between RA content and genotoxicity, indicating their possible involvement; however further research is needed to identify and confirm the compounds involved and to elucidate possible genotoxic effects of RAs.

## 1. Introduction

Eutrophication is nowadays pervasive in numerous lakes, rivers, and water reservoirs around the world due to anthropogenic input of nutrients, which create favorable conditions for cyanobacterial and algal mass development [1,2]. Massive cyanobacterial proliferation (blooms) and algal growth in water bodies is additionally enhanced by increased temperatures and higher concentrations of carbon dioxide in the Earth’s changing climate and atmosphere [3,4]. Cyanobacterial blooms not only have a severe impact on the ecosystem—e.g., changes in biodiversity, light conditions, or oxygen concentrations—but can cause a significant decrease in water quality, since many cyanobacterial species produce odors, noxious compounds, and potent toxins [5,6]. From the cyanobacterial blooms analyzed, 25–75% are reported to be toxic [7,8,9].

Cyanotoxins are a chemically diverse group of bioactive compounds and are traditionally classified into four groups, according to their main target organ, as hepatotoxins, neurotoxins, dermatotoxins and irritants, and general cyanotoxins. There is also evidence that certain cyanotoxins are genotoxic and potentially carcinogenic; however, the mechanisms behind their genotoxic activity are still not completely understood (for review see: [2,4,10]). Among the most studied and widespread cyanotoxins, frequently occurring in freshwater blooms, are the cyclic heptapeptides microcystins (MCs) and the alkaloid cylindrospermopsin (CYN) [11]. The principal mechanism of MC toxicity is the inhibition of protein phosphatases, which leads to the hyperphosphorylation of cellular proteins, resulting in the disruption of important cellular processes such as cell division or DNA damage repair. MCs induce genotoxic effects indirectly, primarily through the formation of reactive oxygen species, resulting in oxidative stress and oxidative DNA damage (for review see: [12]). Exposure to MCs can potentially lead to the development of liver or colorectal cancers [1,6]. Of the at least 279 MCs variants identified to date [13], microcystin-LR (MC-LR) is the most potent and most frequently occurring MC congener. MC-LR is classified as a possible human carcinogen (Class 2B) by the International Agency for Research on Cancer (IARC) [14]. CYN, on the other hand, is considered as an emerging cyanotoxin, and studies describing its detrimental effects are just beginning to accumulate. The principal mechanism of its toxicity is the irreversible inhibition of protein synthesis [15]. The processes involved in the induction of genotoxic effects, are however, not well understood. The toxin was reported to be genotoxic in various test systems and is thus potentially carcinogenic, and there is strong evidence that it needs metabolic activation by cytochrome P450 enzymes in order to cause DNA damage and to exert genomic instability (for review, see [10,16]).

Complex cyanobacterial-bloom samples however, frequently exert adverse effects that cannot be explained by the presence of known cyanotoxins alone [12,17,18]. For instance, significant mutagenic activity (Ames assay) was reported in bloom extracts containing MC-LR, CYN, and anatoxin-a, but the isolated cyanotoxins or fractionated extracts have shown lower or no mutagenicity [19]. Ding et al. (1999) also observed strong mutagenicity in the Ames assay, induction of DNA damage in primary rat hepatocytes, and micronucleated polychromatic erythrocytes in mice, i.p. injected with extracts from a *Microcystis aeruginosa* dominated bloom that contained MC-LR in a concentration that could not have caused the observed genotoxic effects alone [20]. These and other similar published findings suggest that complex extracts from environmental cyanobacterial samples contain genotoxic compounds other than the known cyanotoxins [21,22]. Cyanobacteria produce a plethora of diverse biologically active substances, our knowledge about their potential human health hazard is however still limited.

One class of such possibly toxic cyanobacterial products could be retinoids, a family of polyisoprenoid lipids that include retinol (vitamin A) and its analogues such as retinal and retinoic acids (RAs). RAs are the most potent natural retinoids. These low molecular weight lipophilic metabolites of retinol, are well known vertebrate-specific “dietary” hormones that have been shown to be produced by pure laboratory cultures of cyanobacteria and algae [23,24] and were detected in environmental blooms [25,26] and water bodies associated with blooms of diverse cyanobacterial species [27]. Environmental retinoids, especially RAs, have been recognized as an increasing concern in the last decade, due to their teratogenic effects and potential role in the development of deformations in aquatic vertebrates (for review see: [28]). Studies on the genotoxic effects of RAs are however rare. All-trans retinoic acid (ATRA) and its steroidal analog EA-4 were reported to induce micronuclei through chromosomal breakage and chromosome delay, indicating their clastogenic activity in vitro in human lymphocytes and C_2_C_12_ mouse cells at μM levels [29]. Retinoids are also known to elicit diverse physiological effects such as controlling cell differentiation, proliferation, apoptosis, and modulating specific premalignant lesions [30,31].

The aims of the present study were (i) to evaluate the cytotoxic and genotoxic activity of complex extracts from laboratory cultures of potentially toxic species of cyanobacteria and algae in the human hepatocellular carcinoma HepG2 cell line; (ii) to identify bioactive compounds (selected cyanotoxins and RAs) present in the extracts; and (iii) to examine potential relationships between toxin and RA content in the extracts and measured cytotoxicity and genotoxicity endpoints. The cyanobacterial species included in the study were chosen with respect to their frequency of occurrence and abundance in environmental freshwater blooms [26,27]. Representative species from several commonly occurring cyanobacterial orders were selected, which were previously found to produce cyanotoxins and/or RAs [23,25]. The algae species *Desmodesmus quadricauda* was selected as a relevant algal representative, since it is one of the most common freshwater algae genera and often dominates algal assemblages. The cytotoxicity of the cyanobacterial and algal extracts was evaluated with the 3-(4,5-dimethylthiazol-2-yl)-2,5-diphenyltetrazolium bromide (MTT) assay, the formation of DNA strand breaks was assessed with the comet assay, while the influence on genomic instability was evaluated with the cytokinesis-block micronucleus (CBMN) cytome assay. The presence and concentrations of the cyanotoxins MC-RR, MC-YR, MC-LR and CYN, and RAs (ATRA and 9cis-RA) were measured with liquid chromatography electrospray ionization mass spectrometry (LC/ESI-MS).

## 2. Results

Extracts included in the present study were prepared from laboratory cultured cyanobacterial species (*Cylindrospermopsis raciborskii*, *Aphanizomenon gracile*, *Microcystis aeruginosa*, *Microcystis viridis*, *Microcystis ichtyoblabe*, *Planktothrix agardhii*, and *Limnothrix redekei*), representing several diferent cyanobacterial orders and the algal species *Desmodesmus quadricauda* (for details see Table 1).

### 2.1. Cytotoxicity of the Cyanobacterial and Algal Extracts 

**Figure 1:**
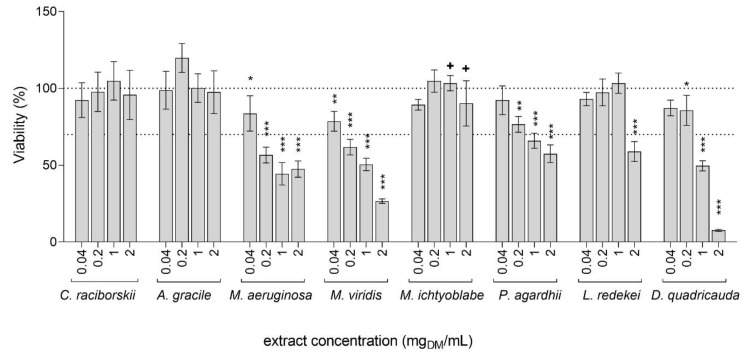
The effect of cyanobacterial and algal extracts on the viability of HepG2 cells. Viability was determined with the MTT assay after the exposure to graded concentrations (0.04, 0.2, 1, and 2 mg_DM_/mL) of extracts for 24 h. Data are represented as percentages of the average OD_570_ of the vehicle control (0.05% methanol). The asterisks denote significant difference from vehicle control (ANOVA, Dunnett’s posttest): * *p* < 0.05, ** *p* < 0.01, *** *p* < 0.001. The cross (+) denotes concentrations where significant morphological changes were observed under the light microscope. The dashed lines represent the 100% and 70% viability thresholds, where the latter indicates the concentration limit used for genotoxicity testing.

The cytotoxicity of the tested extracts was evaluated in HepG2 cells after 24 h of exposure to concentrations ranging from 0.04 to 2 mg_DM_/mL, with the MTT assay. The extracts from *C. raciborskii, A. gracile,* and *M. ichtyoblabe* had no significant influence on HepG2 cell viability, while the other tested extracts reduced cell survival significantly, in a dose-dependent manner (Figure 1). The most cytotoxic were the extracts from *M. viridis* and *M. aeruginosa,* with calculated IC_50_ values of 0.47 and 0.76 mg_DM_/mL, respectively. These extracts significantly reduced cell viability for 21.4% and 16.3%, respectively, already at the lowest tested concentration (0.04 mg_DM_/ml). The extract from *D. quadricauda* was also highly toxic at the higher tested concentrations (1 and 2 mg_DM_/mL), with a reduction in cell viability for 50.4% and 92.4%, respectively. The *L. redekei* extract decreased cell viability for 41.1% at the highest tested concentration (2 mg_DM_/mL), while lower concentrations had no effect. The extract from *P. agardhii* significantly decreased viability for 23.4%, 34.1%, and 42.5% at the concentrations 0.2, 1, and 2 mg_DM_/mL, respectively. Although cell viability decrease was not detected after exposure to the *M. ichtyoblabe* extract with the MTT assay, significant morphologic changes, including cell detachment, were observed at the concentrations 1 and 2 mg_DM_/mL under the light microscope (data not shown). As the MTT assay measures the metabolic activity of the cells, it can give false negative results as a consequence of increased metabolic activity in response to cellular stress, or because at certain conditions, a similar degree of metabolic activity can be maintained also in clearly detached and damaged cells [32]. The *M. ichtyoblabe* extract concentrations that induced morphological changes were therefore also considered as cytotoxic.

### 2.2. Genotoxicity of the Cyanobacterial and Algal Extracts—Comet Assay

**Figure 2:**
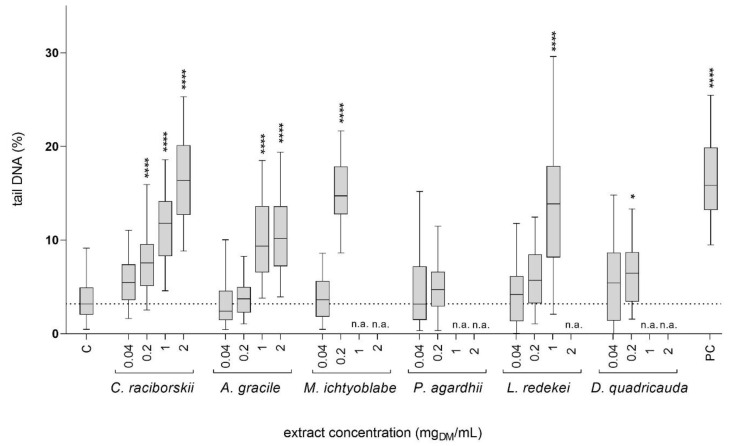
The induction of DNA strand breaks in HepG2 cells after 24 h exposure to graded concentrations (0.04, 0.2, 1, and 2 mg_DM_/mL) of cyanobacterial and algal extracts. Benzo[a]pyrene (PC; 50 µM) was used as a positive control. Fifty cells were analyzed per experimental point in each of the two independent experiments. Data are represented as quantile box plots. The asterisks denote significant difference (nonparametric Kruskal–Wallis, Dunn’s posttest) from the vehicle control (C; 0.05% methanol): * *p* < 0.05, **** *p* < 0.0001. Cytotoxic concentrations of the extracts were not analyzed (n.a). The dotted line denotes the median value of the % tail DNA in vehicle control group.

The induction of DNA strand breaks by the tested cyanobacterial and algal extracts was tested at non-cytotoxic concentrations (viability reduction of less than 30% and no significant morphological changes; Figure 1). After 24 h of exposure, five extracts, *C. raciborskii*, *A. gracile*, *L. redekei*, *M. ichtyoblabe,* and *D. quadricauda* (Figure 2), induced a statistically significant increase in tail DNA. The lowest observed concentration (LOEC) to induce DNA damage for the *C. raciborskii*, *M. ichtyoblabe*, and *D. quadricauda* extracts was 0.2 mg_DM_/mL and 1 mg_DM_/mL for the *A. gracile* and *L. redekei* extracts. The *P. agardhii* extract did not induce DNA damage in HepG2 cells at the tested, non-cytotoxic concentrations.

### 2.3. Assessment of Genomic Instability Induced by the Cyanobacterial and Algal Extracts—CBMN Assay

The influence of the tested extracts on the genomic instability of HepG2 cells was studied with the CBMN assay at concentration that induced less than 55% reduction in cell viability (0.2 mg_DM_/mL). At the tested concentration, none of the extracts reduced the nuclear division index (NDI). None of the tested extracts increased the frequency of micronuclei (MNi) formation, compared to the vehicle control (Table 2). The positive control, etoposide (ET, l μg/mL) induced a significant increase in MNi formation and a decrease in the NDI.

### 2.4. Chemical Analyses

Chemical analyses of the cyanobacterial and algal extracts identified MCs and RAs in most analyzed extracts (Table 3). CYN was not detected in any of the studied extracts [limit of detection (LOD) for CYN was 0.25 ng/g_DM_]. The concentrations of individual MCs (MC-RR, MC-YR, MC-LR) ranged from below LOD (<0.6, <2.5, <10, respectively) to 6.8 × 10^4^ ng/g_DM_. In two extracts (*M. aeruginosa* and *P. agardhii*), MCs were detected at concentrations that were several orders of magnitude higher than in the rest of the extracts. The *M. aeruginosa* biomass contained 6.8 × 10^4^ ng/g_DM_ of MC-LR, and *P. agardhii* biomass contained 1.08 × 10^4^ ng/g_DM_ of MC-YR. The remaining extracts contained less than 300 ng/g_DM_ of the analyzed MCs in total. MC-RR (728.8 ng/g_DM_) was detected in all three *Microcystis* species, MC-YR (12.8–1.08 × 10^4^ ng/g_DM_) in *M. viridis* and *P. agardhii*, and MC-LR (7.5–6.8 × 10^4^) in all three *Microcystis* species and in *P. agardhii*.

RAs were detected in all extracts except those from *M. viridis* and *P. agardhii* (Table 3) with LODs for ATRA and 9cis-RA being 7.5 and 5 ng/g_DM_, respectively. Detected concentrations of ATRA were higher than concentration of 9cis-RA, ranging from 40 up to 1450 ng/g_DM_ (*L. redekei*). 9cis-RA was detected only in the extracts from *C. raciborskii*, *M. aeruginosa*, *M. ichtyoblabe*, and *D. quadricauda* at concentrations from 5.5 to 55 ng/g_DM_ (*M. ichtyoblabe*). 

### 2.5. Assessment of Potential Relationships between MC/RA Content and the Observed Cytotoxic/Genotoxic Effects

Associations between MC or RA content in the extracts and cytotoxicity or genotoxicity endpoints were calculated using linear regression (Table 4). No association could be found between MC-LR and combined MC (MC-LR + MC-RR + MC-YR) content in the extracts and cytotoxicity (average % of viability). There was not enough data to calculate the relationships between MC-RR and MC-YR content and cytotoxicity, as only two extracts, included in the calculations, contained these toxins. Also the association between MC (MC-LR, MC-RR, and MC-YR) content and genotoxicity could not be calculated, because only one genotoxic extract contained MCs.

**Figure 3:**
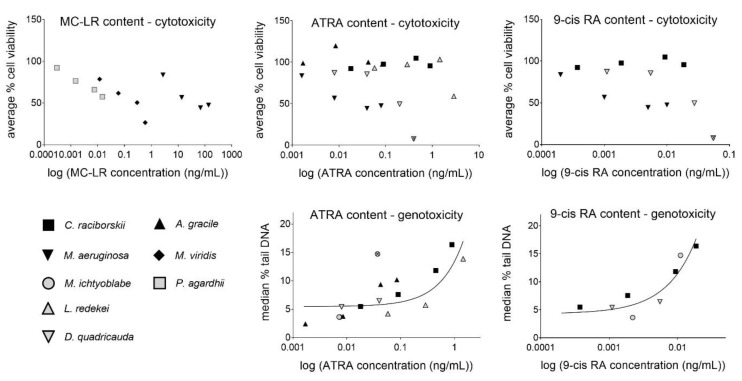
Linear association between MC-LR, ATRA, and 9cis-RA concentration in the extract (ng/mL) and extract cytotoxicity—average % viability (*C. raciborskii*, *A. gracile*, *M. aeruginosa*, *M.viridis*, *P. agardhii*, *L. redekei*, and *D. quadricauda*) and genotoxicity—median % tail DNA (*C. raciborskii*, *A. gracile*, *M. ichtyoblabe*, *L. redekei*, and *D. quadricauda*) in HepG2 cells, after 24 h of exposure. The *M. ichtyoblabe* extract data were excluded from the cytotoxicity assessment, as this extract produced false negative MTT results. Only non-cytotoxic (reduction of viability for less than 30%, and no observed morphological changes) concentrations were included in the genotoxicity correlation calculations. Single outliers or influential points were eliminated from the regression analysis and are marked with x.

There was no significant association between RA content and cytotoxicity. However, significant linear associations were found for RA content (ATRA, 9cis-RA and total RAs (ATRA + 9cis-RA)) and the median % of tail DNA, determined with the comet assay (Table 4, Figure 3). The strongest linear relationship was found for 9cis-RA content in the extracts and DNA damage induction (median % tail DNA) after exposure of the cells to the extracts of *C. raciborskii*, *M. ichtyoblabe*, and *D. quadricauda*. 

## 3. Discussion 

Studies on the adverse health effects of potential harmful environmental exposures have historically focused on effects caused by single compounds. However, exposure to single compounds is not realistic. Complex mixtures of various compounds are present in the environment and exposure to such mixtures can exert significantly different effects compared to single substances, due to various unknown antagonistic, additive or synergistic interactions between chemicals [33,34,35]. Accumulating data supporting this, caused a shift of interest towards environmental mixtures research, representing real life-relevant exposure scenarios. Nevertheless, risk assessment of complex mixtures remains a major challenge due to remaining limitations of the analytical approaches, mixture variability, and scarcely available data. In the present study, the cyto/genotoxic effects of extracts from the cyanobacteria *C. raciborskii, A. gracile*, *M. aeruginosa, M. viridis*, *M. ichtyoblabe*, *P. agardhii*, and *L. redekei,* and the algae *D. quadricauda* (Table 1) were investigated in the human hepatocellular carcinoma cell line, HepG2. The liver is the target organ of many cyanotoxins, including MCs and CYN, and is the center of xenobiotic metabolism in the body. As the HepG2 cell line retained several phase I and II enzymes involved in xenobiotic metabolism and detoxification it is one of the test systems of choice in the fields of toxicology and genetic toxicology [36]. Our results show that complex cyanobacterial extracts from a diverse group of cyanobacterial species, and the algae *D. quadricauda*, can induce cytotoxic (Figure 1) and genotoxic (Figure 2) effects in HepG2 cells. Cyanobacteria produce a plethora of bioactive substances and chemically extremely diverse secondary metabolites. The extracts in our study were therefore complex mixtures, containing in addition to the analyzed MCs (MC-LR, MC-RR, and MC-YR), CYN and RAs (ATRA and 9cis-RA), a wide range of other compounds, possibly including potentially harmful bioactive compounds and other toxins. The observed cytotoxic effects were most probably the consequence of the simultaneous activity of a number of compounds and chemical interactions in the complex mixtures.

Only two extracts, from *C. raciborskii* and *A. gracile*, were not cytotoxic for HepG2 cells at the tested concentrations. The three most cytotoxic cyanobacterial extracts (*M. aeruginosa*, *M. viridis*, and *P. agardhii*) contained the highest concentration of MCs detected in this study, however no significant linear association was found between the combined MC content in the extracts and cytotoxicity. The IC_50_ value for MC-LR in HepG2 cells was reported to be 131 mg/L [37]. No cytotoxicity was reported for MC-LR and MC-RR after 24 h exposure of HepG2 cells to concentrations of 0.1–10 mg/L [38]. The IC_50_ of the *M. aeruginosa* extract in our study was calculated at 0.76 mg_DM_/mL, which corresponds to a MC-LR content of 51.7 µg/L and MC-RR content of 17.5 ng/L. The *M. viridis* extract had an even lower IC_50_ value. It contained significantly lower amounts of MC-LR, but in addition to MC-LR and MC-RR contained also MC-YR. The combined MC content was still much lower than in the *M. aeruginosa* extract. In addition to MCs, we also analyzed the presence of CYN in the extracts. However, concentrations of CYN were below LOD (<0.25 ng/g_DM_) in all of the extracts. Therefore, the analyzed toxins may have contributed to, but were most probably not the sole cause of the observed, relatively high, cytotoxicity of the extracts.

Furthermore, the analyzed RAs (ATRA and 9cis-RA) were present in low concentrations or under LOD in the most cytotoxic extracts. A weak association was found for 9cis-RA content in the extracts and cytotoxicity, which however, considering the low degree (R^2^ < 0.5), probably bears no biological importance. ATRA was previously reported not to induce significant cytotoxicity in HepG2 cells at concentrations up to 300 mg/L, measured with the MTT assay [39]. Recently however, ATRA was shown to induce autophagy and apoptosis in HepG2 cells at 3 mg/L [40]. Nevertheless, the highest content of ATRA in our study was only 1.45 µg/L in the *L. redekei* extract. The direct contribution of 9cis-RA to the extracts’ cytotoxicity was probably non-significant, since this compound exerts a similar toxicity as ATRA [41], but its content in the extracts was about two orders of magnitude lower than the detected ATRA concentrations. It has to be considered that other RAs (not analyzed) could have been present in the extracts, and that the concentrations of total RAs, with similar potencies, could have been much higher. It was reported that besides ATRA, 4-keto ATRA and retinal are most commonly present in high amounts in biomass samples from some cyanobacterial species and various environmental blooms, significantly increasing the combined retinoid-like activities of the extracts [24,25,27].

The genotoxic activity of the extracts was tested after 24 h of exposure of HepG2 cells, to non-cytotoxic concentrations of the extracts, with the comet and the CBMN assays. At the tested concentration (0.2 mg_DM_/mL) none of the extracts induced MNi formation in CBMN assay. Similar results were reported by Abramsson-Zetterberg et al. (2010) [42]. No MNi induction was observed in isolated human lymphocytes exposed to pure MC-LR (75 µg/L) and cyanobacterial extracts (up to 2 mg_DM_/mL), prepared from bloom samples collected from four different lakes in Sweden, containing MC-LR, MC-YR, and MC-RR. Pure MC-LR was reported to increase MNi formation in several different test systems including HepG2 cells; however, at much higher concentrations than detected in our study (≥ 5 mg/L) (for review see: [12]). The genotoxicity of retinoids was described in only one published in vitro study on ATRA and its steroidal analog EA-4. Both were reported to induce MNi in human lymphocytes and murine myoblasts at μM levels and higher [29]. Concentrations of the analyzed retinoids in our samples were again four orders of magnitude lower than ATRA levels reported to induce MNi. 

Five of the six tested extracts in our study statistically significantly induced DNA strand breaks in the comet assay. The highest DNA damage induction was observed after exposure of the cells to the extracts of *C. raciborskii*, *M. ichtyoblabe*, and *D. quadricauda* with a LOEC of 0.2 mg_DM_/mL. The LOEC for the *L. redekei* and *A. gracile* extract was at 1 mg_DM_/mL. Among the extracts analyzed in the comet assay, only one of the genotoxic extracts (*M. ichtyoblabe*) contained MCs (8.7 ng/g_DM_ of MC-RR and 50.5 ng/g_DM_ MC-LR). There is extensive evidence for the genotoxicity of pure cyanotoxins, mainly MCs and CYN (for review see: [10,12,43]). MC-LR induces DNA strand breaks and exerts clastogenic effects in mammalian cells in vitro (for review see: [12,44]). However, the described genotoxic effects are caused by concentrations of MC-LR in the mg/L range [45,46], whereas the concentration of MC-LR detected in our genotoxic sample was significantly lower (10.1 ng/L in the 0.2 mg_DM_/mL *M. ichtyoblabe* extract dilution). Moreover, it was shown that DNA strand breaks caused by MC-LR exposure are transient, reaching a maximum level after 4 h of exposure in HepG2 cells and declining with further exposure, with no detectable increase in DNA strand breaks after 24 h [45], the time point of analysis in our study. Data on in vitro genotoxicity of the other MCs are rare. Also in the genotoxic samples, a significant amount of MC-RR was present only in the *M. ichtyoblabe* extract (containing 1.7 ng/L MC-RR in the 0.2 mg_DM_/mL extract dilution), while significantly higher amount of MC-YR was present in the *P. agardhii* extract (2.2 µg/L MC-YR in the 0.2 mg_DM_/mL extract dilution), where no induction of DNA strand breaks was detected. Our results therefore indicate that the analyzed toxins MC-LR, MC-YR, MC-RR, and CYN, were not a significant cause of the genotoxic effects caused by the extracts, as also CYN was under LOD in all of the samples.

There are other published data showing no correlation between MC-LR content and genotoxicity of cyanobacterial extracts. Ding et al. (1999) observed dose-dependent effects in the comet assay in primary rat hepatocytes after 4 h of exposure to *M. aeruginosa* dominated cyanobacterial bloom extracts, containing ≥ 0.56 mg/L of MC-LR [20]. This is about two orders of magnitude lower compared to effective concentrations of pure MC-LR [45,46], but still, about two orders of magnitude higher than the concentration of MC-LR in our genotoxic sample. Similarly, Sieroslawska (2013) observed mutagenic effects caused by extracts from environmental cyanobacterial samples in the Ames assay, again without any correlation between mutagenicity and content of MC-LR, CYN, and anatoxin-a in the samples, as neither pure MC-LR, CYN nor anatoxin-a give positive results in the Ames assay [19]. The observed adverse effects caused by the cyanobacterial and algal extracts in the present study were most probably the consequence of the interactions between cyanotoxins and other bioactive substances resulting in synergistic or potentiated effects [19,47]. The presence of genotoxic compounds other than the analyzed toxins and/or unknown molecular interactions in the studied extracts would explain the significant genotoxicity observed. The genotoxic cyanobacterial extracts from *C. raciborskii, A. gracile,* and *L. redekei* did not contain any of the analyzed MCs or CYN. In addition, the algal extract from *D. quadricauda* induced DNA strand breaks; however, no toxin production has been described in this species yet. All of the genotoxic extracts in our study contained a significant content of RAs, while in the non-genotoxic extract (*P. agardhii*) the analyzed RAs were below LOD. Significant linear associations were found between ATRA, 9cis-RA, and combined RAs (ATRA + 9cis-RA) content, and induction of DNA strand breaks. The highest degree of linear association with the genotoxicity endpoint was observed for 9cis-RA content in the extracts (linear model: R^2^ = 0.88). We still have to bear in mind that there might be other genotoxic substances in the extracts that somehow correlate with the analyzed RAs in the different species. Still, our results indicate possible involvement of the analyzed RAs in the genotoxicity of the extracts. While due to their relatively low concentrations in the extracts, they could probably not have directly caused the observed effects alone, but rather in combination with other retinoids and/or interactions with cyanotoxins and/or the multitude of other substances probably present in the extracts. The results of the present study highlight the importance of studying complex mixtures as they can exert effects that are significantly different from those caused by pure substances, due to various unknown antagonistic, additive, or synergistic interactions between chemicals [33,34,35]. 

## 4. Conclusions

Cyanobacteria and algae produce a vast diversity of bioactive compounds that can be simultaneously present in the environment. Consequently, real life exposure scenarios are those to complex mixtures that can induce additive, synergistic or antagonistic effects due to chemical interactions between the components. It is therefore important to characterize the toxic effects of complex mixtures as these can differ substantially from those induced by pure compounds. The present study describes cytotoxic and genotoxic effects of complex biomass extracts from cultivated cyanobacteria (*C. raciborskii*, *A. gracile*, *L. redekei*, *M. aeruginosa*, *M. viridis*, *M. ichtyoblabe*, *P. agardhii*) and the alga *D. quadricauda*. Six out of eight extracts were cytotoxic and five induced DNA strand breaks at non-cytotoxic concentrations in HepG2 cells. Due to the low concentration of MCs and CYN detected in the genotoxic extracts, the observed DNA strand breaks could not have been caused by these toxins. RAs (ATRA and 9cis-RA) were on the other hand detected in all of the genotoxic extracts. It has to be emphasized that other unidentified bioactive compounds were most probably present in the studied extracts, and that the observed effects were most likely the result of the simultaneous action of a complex mixture of biologically active compounds.

## 5. Materials and Methods

### 5.1. Cyanobacterial Cultures and Extraction

Complex biomass extracts used in the present study were prepared from laboratory cultured cyanobacterial and algal species (Table 1). For each sample, 100 mg of freeze-dried biomass was gradually extracted in a glass test tube. Initially, 4 mL of 100% methanol was added and cells were disintegrated by sonication with an ultrasonic disintegrator (100% power, cycle 0.9; Sonopuls HD 2070, BANDELIN electronic GmbH & Co. KG: Berlin, Germany) twice for 2 min at 4 °C. Then, the sample was centrifuged (3750 g, 5 min) to remove cellular debris. Supernatant was transferred to a glass vial, and the pellet was washed by vortexing (15 s) in additional 1 mL of fresh methanol, and again centrifuged (3750 g, 5 min). The obtained second supernatant was merged with the first supernatant. After that, 4 mL of fresh methanol was mixed with the pellet, sonicated (5 s), and the tube was shaken (100 rpm) on an orbital shaker(GFL 3020, GENEO BioTechProducts GmbH: Hamburg, Germany). After 2 h of shaking, the sample was centrifuged (3750 g, 5 min), and the obtained supernatant was merged with the previous supernatants. The pellet was washed by vortexing (15 s) in additional 1 mL of fresh methanol, centrifuged (3750 g, 5 min), and the obtained supernatant was added to the previous supernatants. The final extract (the mixture of supernatants) was evaporated to the volume of 0.25 mL under the stream of nitrogen to reach the concentration of 400 g_DM_/L (DM—dry mass). 

### 5.2. Cell Culture and Treatment

HepG2 cells were purchased from the American type culture collection (ATCC, USA). The cells were cultivated in Minimum Essential Medium (MEM) containing 10% FBS, 2 mM L-glutamine, 100 U/ml penicillin/streptomycin and 0.5 mM Non-Essential Amino Acid Solution (all from Sigma, St. Louis MO, USA) at 37 °C in 5% CO_2_ atmosphere. Prior to the treatment, the cells were seeded onto 96-well plates for the MTT assay (density 8 × 10^3^ cells/well), 12-well tissue culture treated plates for the comet assay (density 8 × 10^4^ cells/well) and 25 cm^2^ culture flask (all Corning Costar Corporation, USA) for the CBMN assay (density 5 × 10^5^ cells/flask) and incubated for 24 h to attach. The medium was then replaced with fresh complete medium containing graded concentrations of the cyanobacterial and algal extracts and incubated at 37 °C in 5% CO_2_ humidified atmosphere. In all experiments, methanol (MeOH; 0.05%) was used as the vehicle control.

### 5.3. Cytotoxicity Assay 

The cytotoxicity of the extracts was determined with 3-(4,5-dimethylthiazol-2-yl)-2,5-diphenyltetrazolium bromide (MTT) as described by [35]. This assay measures the reduction of MTT to insoluble formazan in metabolically active cells. After 24 h exposure of HepG2 cells to the extracts (0.04, 0.2, 1, and 2 mg_DM_/mL), MTT was added at a final concentration of 0.5 mg/mL and the cells were further incubated for 3 h at 37 °C. Subsequently, the medium was removed and the formed formazan crystals were dissolved in DMSO. Each concentration of each extract was tested in five replicates. The optical density (OD) was measured at 570 nm (reference filter 690 nm) using a spectrophotometer (Synergy MX, Biotek, Winooski VT, USA). Cell viability was determined by comparing the measured OD values of the test samples to OD values of the vehicle control. IC_50_ values were calculated using GraphPad Prism 9.0.0 software (GraphPad Software Inc., San Diego CA, USA). In parallel to the MTT viability assay, morphologic changes of the cells were evaluated under the light microscope (data not shown).

### 5.4. Comet Assay

HepG2 cells were exposed to non-cytotoxic concentrations of the extracts (0.04, 0.2, 1 and 2 mg_DM_/mL), for 24 h. Extract concentrations that reduced cell viability by more than 30% [48] or caused significant morphological changes, were considered cytotoxic and were not analyzed in the comet assay. As the extracts of *M. aeruginosa* and *M. viridis* were highly cytotoxic, these extracts were not tested in the comet assay. Benzo[a]pyrene (B[a]P; 50 μM), a well-established pro-carcinogen that needs metabolic activation by cytochrome P450 enzymes, was used as the positive control. At the end of the exposure, the comet assay was performed according to Žegura and Filipič (2004) [46]. Briefly, 30 µL of cell suspension was mixed with 70 µL 1% LMP agarose and added on the surface of fully frosted slides pre-coated with 1% NMP agarose (80 µL). Subsequently, the cells were lysed (100 mM EDTA, 2.5 M NaCl, 1% Triton X-100, pH 10; 4 °C) for 1 h, then placed into an alkaline solution (300 mM NaOH, 1 mM EDTANa_2_, pH 13, 4 °C) for 20 min to allow DNA unwinding, and electrophoresed for 20 min at 25 V (300 mA). Finally, the slides were neutralized in 0.4 M Tris buffer (pH 7.5) for 15 min, stained with ethidium bromide (5 μg/mL) and analyzed using a fluorescence microscope (Nikon, Eclipse 800, Minato City, Tokyo, Japan). For each experimental point images of 50 randomly selected nuclei were analyzed with the image analysis software Comet Assay IV (Perceptive Instruments, Bury St Edmunds, UK). For each sample, two independent experiments were performed (each in duplicate) and the results are expressed as % of DNA in the comet tail.

### 5.5. Cytokinesis-Block Micronucleus (CBMN) Cytome Assay

HepG2 cells were exposed to the extracts at the concentration of 0.2 mg_DM_/mL for 24 h. At the tested concentration none of the extracts decreased cell viability for more than 55% as recommended by the OECD Test Guideline No. 487 [49]. The anti-cancer drug etoposide (ET, 1 μg/mL) was used as the positive control as it is known to induce clastogenic effects in HepG2 cells. After the treatment, the cells were washed twice with PBS buffer, the medium containing cytochalasin B (2 μg/mL) was added, and the cells were incubated for additional 26 h at 37 °C in 5% CO_2_ humidified atmosphere. The medium containing cytochalasin B was collected in a centrifuge tube, the cells were washed with PBS buffer, harvested, and added to the corresponding centrifuge tube. The slides were prepared as described by Štraser et al. (2011) [50]. For the analysis, the slides were stained with acridine orange (20 μg/mL) and examined under the fluorescence microscope (Nikon, Eclipse 800, Minato City, Tokyo, Japan). For each experimental point MNi were counted in 1000 BNC at 400× magnification according to the criteria published by Fenech (2000) [51]. The NDI was estimated by scoring 500 cells with one to four nuclei and calculated using the formula [M1 + 2M2 + 3(M3 + M4)]/500, where M1, M2, M3, and M4 represent the number of cells with one to four nuclei, respectively. The experiments were repeated three times independently.

### 5.6. Chemical Analyses of Toxins and RAs in the Extracts

#### 5.6.1. Analyses of Microcystins 

All chemical analyses were conducted using liquid chromatography electrospray ionization mass spectrometry (LC/ESI-MS). Analyses of microcystins (MCs) were performed with an Agilent 1290 series HPLC (Agilent Technologies, Germany) consisting of a vacuum degasser, a binary pump, an autosampler, and a thermostatted column compartment kept at 30 °C. The column was a Phenomenex LUNA C18 end-capped (3 mm) 100 × 2 mm i.d., equipped with a Phenomenex SecureGuard C18 guard column (Phenomenex, Torrance CA, USA). The mobile phase consisted of 5 mM ammonium acetate in water, pH 4 (A) and methanol-acetonitrile mixture (1:1) with 5 mM ammonium acetate (B). The binary pump gradient was non-linear (increase from 25% B at 0 min to 80% B at 2 min, then increase to 95% B at 10 min, then 95% B for 4 min and 4 min column equilibration to initial conditions (25% B); the flow rate was 0.25 mL/min. Five µL per individual sample was injected for the analyses. The mass spectrometer AB Sciex Qtrap 5500 (AB Sciex, Concord, Redwood City CA, USA) with electrospray ionization (ESI) was used for detection. Ions were detected in the positive mode. The ionization parameters were as follows: capillary voltage, 5.5 kV; desolvation temperature, 350 °C; cone gas 150 L/h, drying gas 600 L/h, nebulizer 7.0 bar. In scheduled MRM mode the following *m/z* transitions were monitored (with corresponding values of declustering potential-DP (V), entrance potential-EP (V) and collision energy-CE (V)): MC-RR 519.8 > 135.1 (DP 156, EP 10, CE 37) and 102.9 (DP 156, EP 10, CE 91), MC-YR 1045.5 > 102.9 (DP 241, EP 10, CE 129) and 212.9 (DP 241, EP 10, CE 69), MC-LR 995.5 > 102.9 (DP 171, EP 10, CE 129) and 105.1 (DP 171, EP 10, CE 127). The quantification of analysts was based on external standards of MC-RR, MC-YR, and MC-LR (Enzo Life Sciences, Inc., Farmingdale NY, USA).

#### 5.6.2. Analyses of Cylindrospermopsin

Analyses of cylindrospermopsin (CYN) were performed with a Waters Acquity HPLC (Waters, UK) consisting of a vacuum degasser, a binary pump, an autosampler, and a thermostatted column compartment kept at 30 °C. The column was Supelco Ascentis RP-Amide, 3.0 µm, 100 × 2.1 mm, equipped with Phenomenex SecureGuard C18 guard column. The mobile phase consisted of 0.1% formic acid in water (A) and 0.1% formic acid in methanol (B). The binary pump gradient was non-linear (0% B for one minute, then increase from 0% B at 1 min to 60% B at 3 min, then 60% B for 2 min, then increase to 90% B at 6 min, then 90% B for 1 min and 5 min column equilibration to initial conditions 0% B). The flow rate was 0.25 mL/min. Five μL per individual sample was injected for the analyses. Mass spectrometer used for detection was Waters XEVO TQ-S with electrospray ionization (ESI). Ions were detected in the positive mode. The ionization parameters were as follows: capillary voltage, 5.5 kV; desolvation temperature, 350 °C; curtain gas 20 psi, drying gas 40 psi, nebulizer 30 psi. In scheduled MRM mode the following *m/z* transitions were monitored (with corresponding values of declustering-DP (V), entrance potential-EP (V) and collision energy-CE (V)): CYN 415.9 > 336.1 (DP 121, EP 10, CE 31) and 194.1 (DP 121, EP 10, CE 47). CYN was quantified using external calibration.

#### 5.6.3. Analyses of Retinoic Acids 

Analyses of retinoic acids were performed with Waters Acquity HPLC consisting of a vacuum degasser, a binary pump, an autosampler, and a thermostatted column compartment kept at 40 °C. The column was Waters Acquity UPLC BEH C18, 1.7 mm, 100 × 2.1 mm i.d., equipped with a Waters Acquity UPLC BEH C18 VanGuard Pre-column. The mobile phase consisted of 0.1% formic acid in water (A) and 0.1% formic acid in acetonitrile (B). The binary pump gradient was non-linear (increase from 20% B at 0 min to 70% B at 1 min, then increase to 100% B at 5 min, then 100% B for 1 min and 2 min column equilibration to initial conditions 20% B). The flow rate was 0.3 mL/min. Five µL per individual sample was injected for analysis. The mass spectrometer used for detection was Waters XEVO TQ-S with electrospray ionization (ESI). Ions were detected in the positive mode. The ionization parameters were as follows: capillary voltage, 1.0 kV; desolvation temperature, 450 °C; cone gas 150 L/h, drying gas 600 L/h, nebulizer 7.0 bar. In scheduled MRM mode the following *m/z* transitions were monitored (with corresponding values of cone voltage-CV (V), source offset-SO (V) and collision energy-CE (V)): ATRA 301.2 > 205.2 (CV 30, SO 60, CE 12) and 159.1 (CV 30, SO 60, CE 23), 9 cis-RA 301.2 > 205.2 (CV 30, SO 60, CE 13) and 159.1 (CV 30, SO 60, CE 23). The quantification of analytes was based on external standards of ATRA and 9cis-RA.

### 5.7. Statistics 

The statistical analyses were performed by one-way analysis of variance (ANOVA) for the MTT and CBMN assays, and by the non-parametric Kruskal–Wallis test for the comet assay, in the GraphPad Prism 9.0.0 software (GraphPad Software Inc., San Diego CA, USA). Posttests, the Dunnett’s test (ANOVA posttest) and the Dunn’s test (Kruskal-Wallis posttest), were used for multiple comparisons of the results from samples versus the vehicle control; *p* < 0.05 was considered as statistically significant. 

The linear association between toxin and RA content in the extracts and cytotoxicity (average % viability) and genotoxicity (median tail DNA %) endpoints were calculated using linear regression in GraphPad Prism 9.0.0. MC and RA content in the extracts and extract dilutions, was calculated based on the results from the chemical analysis. Data points with a concentration of the corresponding toxins or RAs under the LOD were not included in the linear regression analyisis. Single outliers or single higly influential points detected in GraphPad Prism 9.0.0 and were eliminated. For the cytotoxicity regression analysis, all data were included, except for the *M. ichtyoblabe* extract, which gave false negative results in the MTT assay. For the genotoxicity regression calculations, only non-cytotoxic concentrations (same criteria as for the comet assay) were used in the calculation. The correlations were calculated for variables where at least 9 or more data points from at least three different cyanobacterial species were available.

## Figures and Tables

**Table 1:** Cyanobacterial and algal species used in the study.

Species	Order	Source	Place of Origin
*Cylindrospermopsis raciborskii*	Nostocales	SAG 1.97	Lake Balaton, Hungary
*Aphanizomenon gracile*	Nostocales	RCX 06	Lough Neah, Ireland
*Microcystis aeruginosa*	Chroococcales	PCC 7806	Braakman Reservoir, The Netherlands
*Microcystis viridis*	Chroococcales	PC II21LiP10/IX	Lipno, Czech Republic
*Microcystis ichtyoblabe*	Chroococcales	PC II42LiP10/V	Lipno, Czech Republic
*Planktothrix agardhii*	Oscillatoriales	CCALA 159	Plon lake, Germany
*Limnothrix redekei*	Synechococcales	SAG 3.89	Edebergsee at Plön, Germany
*Desmodesmus quadricauda*	Sphaeropleales	CCALA 463	Greifswald, Germany

Culture collection: SAG—Culture Collection of Algae at the University of Goettingen, RCX—species originates from CCALA 008, but has been long-term cultivated at RECETOX labs, PCC—The Pasteur Culture Collection of Cyanobacteria, PC—personal culture collection of E. Kozlíková-Zapomělová, Biology Centre of the Czech Academy of Sciences, Institute of Hydrobiology, České Budějovice, Czech Republic, CCALA—Culture Collection of Autotrophic Organisms.

**Table 2:** Binucleated cells (BNC) containing micronuclei (MNed) and nuclear division index (NDI) in HepG2 cells exposed to the cyanobacterial and algal extracts (0.2 mg_DM_/mL), and positive control (Etoposide, l μg/mL) for 24 h.

Sample	MNed Cells/10^3^ BNC	Fold Induction	NDI
*C. raciborskii*	32.0 ± 4.0	1.07	1.8 ± 0.04
*A. gracile*	32.7 ± 4.9	1.09	1.8 ± 0.1
*M. aeruginosa*	40.0 ± 10.8	1.33	1.7 ± 0.1
*M. viridis*	33.3 ± 2.1	1.11	1.8 ± 0.1
*M. ichtyoblabe*	30.7 ± 9.5	1.02	1.7 ± 0.1
*P. agardhii*	32.7 ± 10.1	1.09	1.8 ± 0.01
*L. redekei*	35.3 ± 7.2	1.18	1.8 ± 0.1
*D. quadricauda*	31.7 ± 3.5	1.06	1.8 ± 0.04
Vehicle control	30.0 ± 2.0	1	1.8 ± 0.1
Etoposide (l μg/mL)	199.7 ± 35.5 *	6.65	1.2 ± 0.1 *

* significant difference from the vehicle control—One-way ANOVA with Dunnett’s post test; *p* < 0.05.

**Table 3:** Concentrations of microcystins (MCs), cylindrospermopsin (CYN), and retinoic acids (RAs) in cyanobacterial and algal extracts.

Sample	Microcystins (ng/g_DM_)			Cylindrospermopsin (ng/g_DM_)	Retinoic Acids (ng/g_DM_)	
	MC-RR	MC-YR	MC-LR		ATRA	9cis-RA
*C. raciborskii*	<0.6	<2.5	<10	<0.25	450	9.3
*A. gracile*	<0.6	<2.5	<10	<0.25	42.5	<5
*M. aeruginosa*	23.65	<2.5	68,000	<0.25	40	5.5
*M. viridis*	28.8	12.8	295.5	<0.25	<7.5	<5
*M. ichtyoblabe*	8.7	<2.5	50.5	<0.25	180	55
*P. agardhii*	<0.6	10,750	7.5	<0.25	<7.5	<5
*L. redekei*	<0.6	<2.5	<10	<0.25	1450	<5
*D. quadricauda*	<0.6	<2.5	<10	<0.25	200	27.5

**Table 4:** Linear regression for MC/RA content and cytotoxic/genotoxic effects of the extracts, observed in HepG2 cells after 24 h of exposure. The endpoints assessed were the average % of viability for cytotoxicity and the median value of the % of tail DNA for genotoxicity.

Linear Regression	Cytotoxicity (Average % Viability)			Genotoxicity (Median Tail DNA%)		
	R^2^	*p*	n	R^2^	*p*	n
ATRA content	0.02	0.5686	20	**0.63**	**0.0004**	**15**
9cis-RA content	0.10	0.3253	12	**0.88**	**0.0002**	**9**
Combined RAs content	0.02	0.5584	20	**0.64**	**0.0004**	**15**
MC-LR content	0.14	0.2011	13	n.a.	n.a.	6
MC-RR content	n.a.	n.a.	9	n.a.	n.a.	2
MC-YR content	n.a	n.a	9	n.a.	n.a.	4
Combined MCs content	0.18	0.1122	13	n.a.	n.a.	6

Bold values denote statistical significance (*p* < 0.001). Insuficient amount of data, not analyzed (n.a.).
